# Cognitive reactivity mediates the relationship between neuroticism and depression

**DOI:** 10.1016/j.brat.2009.12.005

**Published:** 2010-04

**Authors:** Thorsten Barnhofer, Tobias Chittka

**Affiliations:** a University of Oxford, Department of Psychiatry, Warneford Hospital, Oxford, OX3 7JX, United Kingdom; b Institute of Clinical Psychology and Psychotherapy, Technische Universität Dresden, Chemnitzer Str. 46, 01187 Dresden, Germany

**Keywords:** Depression, Neuroticism, Rumination, Cognitive reactivity

## Abstract

Although neuroticism has long been established as an important risk factor for depression, the mechanisms through which this temperamental predisposition translates into the occurrence of symptoms are still relatively unclear. This study investigated cognitive reactivity, i.e. the ease with which particular patterns of negative thinking are reactivated in response to mild low mood, as a potential mediator. Individuals with (*N* = 98) and without a previous history of depression (*N* = 83) who had provided neuroticism scores six years previously were assessed for cognitive reactivity and current symptoms of depression using self-report questionnaires. Tendencies to respond to mild low mood with ruminative thinking mediated the relation between neuroticism and current symptoms of depression in both groups. Reactivation of hopelessness and suicidal thinking occurred as an additional mediator only in those with a history of previous depression. The results suggest that neuroticism predisposes individuals to depression by generally increasing the likelihood of ruminative responses to low mood. In those with a history of depression in the past, neuroticism additionally increases risk of recurrence by facilitating reactivation of previously associated patterns such as suicidal thinking and hopelessness. These findings suggest potential targets for interventions to help preventing the occurrence, or recurrence of depression in those who due to their temperamental predisposition are at an increased risk.

## Introduction

Neuroticism is considered to be a temperamental factor that predisposes individuals for a range of emotional psychopathologies and other aversive outcomes (see for example, Clark, Watson, & Mineka, 1994). Research on depression suggests that neuroticism predicts onset of depressive disorders (De Graaf, Bijl, Ravelli, Smit, & Vollebergh, 2002; Kendler, Gatz, Gardner, & Pedersen, 2006; Kendler, Neale, Kessler, Heath, & Eaves, 1993; Ormel, Oldehinkel, & Vollebergh, 2004); that those who are high in neuroticism are likely to suffer from more chronic episodes of depression (Duggan, Lee, & Murray, 1990; Hirschfeld, Klerman, Andreasen, & Clayton, 1986; Rhebergen et al., in press; Weissman, Prusoff, & Klerman, 1978); and that neuroticism modifies the impact of life events, that is the experience of stressful life events is more likely to lead into depression in those who are high in neuroticism than those who are low in neuroticism (Ormel, Oldehinkel, & Brilman, 2001; van Os & Jones, 1999). These are well established and important findings. However, their implications for the understanding and treatment of vulnerability for depression are disappointingly limited (cf. Ormel, Rosmalen, & Farmer, 2004). Neuroticism is defined as a temperamental factor that is presumed to be relatively stable over time, and as such amenable to therapeutic interventions only to a limited degree. Furthermore, the multi-facetted nature of the construct and its overlap with measures of distress themselves have made it difficult to draw conclusions regarding the particular vulnerability mechanisms it indexes. In order to better understand such mechanisms, it would be helpful to learn more about the factors that mediate the relationship between neuroticism and depression, in particular, how neuroticism relates to more proximal, potentially malleable factors that research has already shown to be implicated in vulnerability for depression.

Neuroticism reflects a global dimension of negative emotionality that encompasses the tendencies to experience negative affect in the face of minor stressors, to be aroused quickly and for arousal to fall slowly following stimulation. It also reflects tendencies towards worrying and post-event processing, tendencies to appraise events as stressful and an inability to control urges (Widiger, Hurt, & Frances, 1984). A core feature of neuroticism is a difficulty in emotion regulation. According to Eysenck and Eysenck (1991) someone who is high on N is “overly emotional, reacting too strongly to all sorts of stimuli, and finds it difficult to get back on an even keel after each emotionally arousing experience” (Eysenck & Eysenck, 1991, p. 4). It is easily conceivable how such temperamental features may lay the ground for the development of maladaptive reactions and strategies more specifically related to the occurrence of depression. Recent evidence has particularly highlighted the role of rumination as a factor accounting for the relation between neuroticism and depression (Kuyken, Watkins, Holden, & Cook, 2006; Muris, Roelofs, Rassin, Franken, & Mayer, 2005; Roelofs, Huibers, Peeters, & Arntz, 2008; Roelofs, Huibers, Peeters, Arntz, & van Os, 2008). Rumination has been defined as a “mode of responding to distress that involves repetitively and passively focusing on symptoms of distress and on the possible causes and consequences of these symptoms” (Nolen-Hoeksema, Wisco, & Lyubomirsky, 2008, p. 400). A large body of research has demonstrated relations between rumination and the occurrence of depression (for overviews see Nolen-Hoeksema et al., 2008; Smith & Alloy, 2009). At the same time, rumination may easily arise out of tendencies towards post-event processing. In fact, repetitive thinking, a hallmark of rumination, has been described by some commentators as a cognitive manifestation of neuroticism (Segerstrom, Tsao, Alden, & Craske, 2000). As described above, recent research supports the assumption of a meditational role of rumination (Kuyken et al., 2006; Muris et al., 2005; Roelofs, Huibers, Peeters, & Arntz, 2008; Roelofs, Huibers, Peeters, Arntz, & van Os, 2008). However, the results of these studies also showed that rumination only partially mediated the relation between neuroticism and depression, suggesting that further factors need to be taken into account.

Another process that has been found to be particularly relevant for the understanding of vulnerability to depression is cognitive reactivity. Cognitive reactivity describes the finding that, once they have become established, negative patterns of thinking can easily be reactivated through only minor triggers such as subtle changes in mood. A number of studies have demonstrated this phenomenon in individuals at risk for depression (for overviews see Lau, Segal, & Williams, 2004; Scher, Ingram, & Segal, 2005). Theoretical accounts have taken such findings to suggest that negative patterns of thinking become associated with negative mood during previous experiences of depression, and that these associations then remain latent during times of normal mood (“differential activation” hypothesis, Teasdale, 1988). Prospective research has demonstrated that those who remain reactive during times of recovery are more likely to relapse (Segal, Gemar, & Williams, 1999; Segal et al., 2006). Given that neuroticism is characterized by an increased sensitivity to emotional stimuli, cognitive reactivity may play a particular role in those who are high in neuroticism. It is conceivable that those who are high in neuroticism are at an increased risk to both acquire negative patterns of thinking and experience their reactivation in response to mild triggers. This may be the case especially when individuals have been depressed in the past given that associations between negative thinking and mood are particularly likely to be formed during times when both of them are predominant. The current study was aimed at investigating this mechanism and to look at the degree to which reactivation of particular aspects of negative thinking and rumination mediate the relation between neuroticism and current symptoms of depression in a sample of individuals with and without a history of depression. In order to assess cognitive reactivity, the study used a self-report questionnaire called the Leiden Index of Depression Sensitivity (LEIDS, Van der Does, 2002).

The LEIDS is based on the assumption that important aspects of cognitive reactivity are accessible to self-report. It assesses cognitive reactivity by asking participants to describe how they would feel and think if they were to experience a low mood. The different sub-scales of the questionnaire assess both cognitive processes and contents that may occur as a response to negative mood such as rumination or thoughts relating to hopelessness or thoughts relating to attempts at harm avoidance. Importantly, previous research has shown that the LEIDS not only differentiates between previously depressed and never-depressed samples when they are in normal mood (Moulds, Kandris, Williams, Lang, Yap, & Hoffmeister, 2008; Van der Does, 2005) but also predicts changes in thinking following negative mood induction. In a study assessing cognitive reactivity in individuals with and without previous history of depression, Van der Does (2002) found that LEIDS scores predicted change in dysfunctional attitudes following mood induction. Studies in previously depressed groups with a history of suicidality have found that self-reported cognitive reactivity predicts changes in a cognitive indicator of hopelessness following mood induction (Williams, Van der Does, Barnhofer, Crane, & Segal, 2008) and that reactivity profiles can differentiate between patients with and without suicidal ideation during previous episodes (Antypa, Van der Does, Penninx, in press).

Most of the research so far that has investigated potential cognitive mediators of the relation between neuroticism and depression has relied on cross-sectional assessments (Kuyken et al., 2006; Muris et al., 2005; Roelofs, Huibers, Peeters, & Arntz, 2008; Roelofs, Huibers, Peeters, Arntz, & van Os, 2008). This is problematic as many of the items of neuroticism scales ask about current distress and reactions to distress with only vague time specifiers and assessing neuroticism at the same time as mediator and criterion variables may, therefore, artificially inflate associations (Ormel, Oldehinkel, et al., 2004; Ormel, Rosmalen, et al., 2004). For the current study we were able to investigate a sample in which neuroticism had been assessed 6 years before we re-contacted them, thus allowing investigations of how neuroticism related prospectively to current symptoms of depression and cognitive reactivity. We hypothesized that (1) neuroticism would be positively associated with current symptoms of depression and (2) that cognitive reactivity as assessed by the LEIDS would mediate the relation between neuroticism and current levels of depression in participants both with and without a history of depression. In order to investigate relative contributions of particular patterns of cognitive reactivity, the different sub-scales of the LEIDS were entered simultaneously into a multiple mediator model. Separate models were computed for participants with and without a history of depression and any differences between models followed up by formally testing moderating effects of previous history.

## Method

### Participants

Participants were recruited by contacting individuals who had previously taken part in a study on neuroticism that had established a large randomly-ascertained family cohort in southwest England (*N* = 88.000; Martin et al., 2000) and who had given their written permission to be contacted again for participation in further research. As part of the previous research, individuals had provided information on neuroticism 6 years before the start of the current study. In addition, diagnostic information on previous history of depression was available from answers to a questionnaire screening for Major Depression that contained questions about each of the DSM-IV criteria. This measure had been specifically designed for the initial genetic study. Prior diagnostic status had been derived using the DSM-IV algorithm except that questions referred to a period of 4 weeks rather than 2 weeks making diagnoses generally more conservative. In recruiting participants for the current research an attempt was made to contact comparable numbers of participants with and without a previous history of depression. 707 potential participants were contacted with an initial letter, of which 223 (32%) indicated their willingness to take part and were sent a questionnaire booklet along with an informed consent sheet in a stamped return envelope. One-hundred and eighty-two (81%) individuals returned the questionnaire booklet and filled in the consent form for the study, 98 of them had suffered from a previous episode of depression and 83 had never been depressed in their life. Mean age of the participants at the time they filled in the second set of questionnaires was *M* = 48.03 years (*SD* = 6.88). One-hundred and five (57.7%) of them were women, 77 (42.3%) of them were men. The study received ethical approval from the Oxfordshire Psychiatric Research Ethics Committee.

### Measures

Questionnaires in the booklet sent to participants included the Leiden Index of Depression Sensitivity (LEIDS), the Beck Depression Inventory-II, and the Major Depression Questionnaire (MDQ). Participants were instructed to fill in the questionnaire booklet at a quiet place at their home where they did not expect to be disturbed for approximately 45 min.

Neuroticism scores had been assessed 6 years before as part of a large community based study using commercial mailing in which participants were sent the Eysenck Personality Questionnaire (EPQ) to fill in at home and send back via mail.

#### Eysenck Personality Questionnaire (EPQ)

The EPQ (Eysenck & Eysenck, 1991) is a self-report questionnaire consisting of 90 items with a binary response format. The neuroticism scale of the EPQ consists of 23 items. Internal consistency in the current sample was good (Cronbach's *α* = .94).

#### Leiden Index of Depression Sensitivity (LEIDS)

The LEIDS (Van der Does, 2002) is a self-report measure of cognitive reactivity to sad mood. The current study used a revised version of the questionnaire (Van der Does & Williams, 2003) that contains 34 items. Participants are asked to report to what extent they display particular forms of thinking in response to low mood. In addition to an overall score of cognitive reactivity the LEIDS provides scores on six sub-scales with items assessing reactivation of Hopelessness/Suicidality (*When I feel down, I more often feel hopeless about everything*), Acceptance/Coping (*When I am sad, I feel more like myself*), Aggression (*When I feel down, I lose my temper more easily*), Control/Perfectionism (*When in a sad mood, I become more bothered by perfectionism*); Harm Avoidance (*When I feel down I take fewer risks*), and Rumination (*When I feel sad, I spend more time thinking about the possible causes of my moods*). Internal consistencies in the current study were LEIDS Hopelessness/Suicidality: *α* = .88; LEIDS Acceptance: *α* = .53, LEIDS Aggression: *α* = .77, LEIDS Control/Perfectionism: *α* = .62, LEIDS Harm avoidance: *α* = .65, LEIDS Rumination: *α* = .81.

#### Major Depression Questionnaire (MDQ)

The Major Depression Questionnaire (MDQ, Van der Does, Barnhofer, & Williams, 2003) is a self-report measure designed to derive diagnoses of current and past Major Depression. In a series of questions, it asks participants to report about presence or absence of DSM-IV criteria for current and past major depression (American Psychiatric Association, 1994), including questions on impact on functioning and exclusion criteria such as bereavement. Consistency of diagnoses derived by the questionnaire with diagnoses based on interviews was assessed in a subsample of 39 individuals of the current sample who participated in a face to face interview using the Structured Clinical Interview for DSM-IV (SCID, First, Gibbon, & Spitzer, 2002). MDQ self-ratings correctly identified all of the 19 participants who received a diagnosis based on the interview and 15 out of the 20 participants who did not receive a diagnosis (Sensitivity = 100%, Specificity = 75%).

## Statistical analysis

**Fig. 1:**
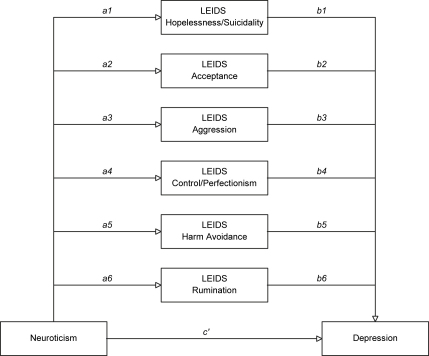
Mediation model depicting direct (weight *c*′) and indirect effects (sum of all *a* × *b* weights) of neuroticism on depression tested in the current study.

Analyses of mediation effects used a multiple mediation model with all LEIDS sub-scales entered simultaneously. Meditation was investigated by directly testing significance of the indirect effect of the independent variable (IV) on the dependent variable (DV) through mediator (M) quantified as the product of the effects of the IV on M, *a*, and the effect of M on DV, partialling out the effect of the IV, *b* (see Fig. 1).

Fig. 1 Following suggestions by Preacher and Hayes (2008) the current study used a bootstrapping approach in which a point estimate of the indirect effect was derived from the mean of the 5000 estimates of *ab* and 95% percentile-based confidence intervals were computed using the cut-offs for the 2.5% highest and lowest scores of the empirical distribution. Indirect effects were considered as significant when the bias corrected and accelerated confidence interval did not include zero.

In order to establish basic relations between variables we first computed first-order correlations between the IV, DV and mediators. Multiple mediator models were then computed separately for participants with and without a history of previous depression. In the case of scales that occurred as mediators in one but not the other group analyses were followed up with moderated mediation models (Preacher, Rucker, & Hayes, 2007) using past history of depression as a moderator in order to formally test for interactions.

## Results

### Group comparisons

Group comparisons showed that those with a history of depression were on average slightly older than those without a history of previous depression (never depressed: *M* = 50.01, *SD* = 6.75; previously depressed: *M* = 46.35, *SD* = 6.59; *t* (179) = 3.69, *p* < .001). As would have been expected, those who had been depressed in the past had significantly higher neuroticism scores as well as significantly higher levels of current symptoms of depression. Previously depressed participants also described themselves as tending to show stronger cognitive reactivity on all sub-scales of the LEIDS apart from the subscale Acceptance/Coping, which assesses a facet of an adaptive way of responding to negative mood. Mean questionnaire scores and standard deviations in the two groups are listed in Table 1.

### Correlational findings

Table 2 shows first-order correlations between neuroticism, current depression and cognitive reactivity scores in the two groups. Neuroticism measured 6 years previously was significantly and positively associated with current levels of depression in both participants with and without a history of depression. Neuroticism was also significantly and positively related to cognitive reactivity in both groups. Significant relations were found for all of the LEIDS sub-scales with the exception of the LEIDS subscale Acceptance/Coping which failed to show a significant relation in the group without a previous history of depression. Furthermore, most of the LEIDS scales were significantly and positively related to current BDI scores. In the never-depressed groups, significant relations between LEIDS and BDI were found for the sub-scales Hopelessness/Suicidality, Aggression, Control/Perfectionism, Harm Avoidance, and Rumination. In the previously depressed group significant relations between LEIDS and BDI were found for the sub-scales Hopelessness/Suicidality, Harm Avoidance and Rumination. The sub-scales of the LEIDS were highly correlated between each other in both groups.

## Mediation analyses

Multiple mediator models in which all of the LEIDS sub-scales were entered simultaneously allowed investigation of the indirect effects of the different LEIDS sub-scales while controlling for the effect of the other scales. Results are summarized in Table 3.

In both groups, total effects (*c*) indicated significant and substantial relations between neuroticism and depression. In the previously depressed group, LEIDS hopelessness/suicidality and LEIDS rumination both significantly mediated the relation between neuroticism and current depression. Partial correlations indicated that LEIDS hopelessness/suicidality and LEIDS rumination accounted for 46 and 39%, respectively, of the variance in depressive symptoms explained by neuroticism. In the never-depressed group only LEIDS rumination emerged as a significant mediator of the relation between neuroticism and depression with partial correlations indicating that LEIDS rumination accounted for 21% of the variance in depressive symptoms explained by the neuroticism in this group. Despite significant mediation, the direct effects (*c*′) remained significant in both groups suggesting that LEIDS sub-scales partially mediated the relations between neuroticism and current depression. While LEIDS rumination emerged as a significant mediator in both groups, LEIDS hopelessness/suicidality only emerged as a significant mediator in the previously depressed group, thus, suggesting that this latter effect was moderated by history of depression.

In order to formally test for moderation, we compared the mediating effects of LEIDS Hopelessness/Suicidality in both groups in a moderated mediation model (following procedures outlined by Preacher & Hayes, 2008) with neuroticism as IV, current depression as DV, LEIDS hopelessness/suicidality as the sole mediating variable and LEIDS rumination as a covariate to control for the significant overlap between the two LEIDS sub-scales. Separate models using 5000 bootstrap resamples were computed for previous history of depression entered as a bivariate moderator of the path from neuroticism to LEIDS Hopelessness/Suicidality (*a*) and the path from LEIDS Hopelessness/Suicidality to current depression (*b*). In the model including LEIDS Hopelessness/Suicidality as a moderator of path *a* (from Neuroticism to LEIDS) results showed significant mediation in the previously depressed group but only marginal in the never-depressed group (previous history of depression: Conditional indirect effect = .12, *SE* = .06, *p* = .02; never depressed: Conditional indirect effect = .05, *SE* = .03, *p* = .06). Similar, but numerically stronger results were found in the model with previous history of depression included as a moderator of path *b* (from LEIDS to Depression: those with previous history of depression: Conditional indirect effect = .14, *SE* .05, *p* = .005; those never depressed: Conditional indirect effect = .002, *SE* = .04, *p* = .94). These results thus suggest that previous depression exerts an influence both on whether the effects of neuroticism are transferred into a tendency for reactivation of hopelessness and suicidal thoughts and whether reactivation of such thinking translates into current symptoms of depression.

## Discussion

Despite an extensive body of research that attests to the role of neuroticism as a predispositional and pathoplastic factor in the development of depressive disorders, relatively little is known about how this temperamental risk factor relates to established vulnerability mechanisms. The current study investigated whether tendencies to respond to mild changes in mood with depressogenic patterns of thinking mediate the relation between neuroticism and current symptoms of depression in individuals with and without a previous history of depression. In both groups neuroticism measured several years previously significantly predicted current symptoms of depression supporting the notion that neuroticism acts as a long term marker of risk for depression.

Mediation analyses showed that in both of the groups, tendencies to respond to mild negative moods with ruminative thinking mediated the relation between neuroticism and current depressive symptoms. Regardless of whether participants had previously suffered from depression, the higher they were in neuroticism, the more they reported responding to mild negative mood by repetitively thinking about the possible causes of this mood, and neglecting more active approaches to coping with difficulties, which, in turn, was related to higher levels of depressive symptoms. This finding is in line with previous reports from cross-sectional studies in both clinical and non-clinical groups that have investigated mediating effects of rumination by using a questionnaire designed to assess habitual tendencies to rumination (Kuyken et al., 2006; Muris et al., 2005; Roelofs, Huibers, Peeters, & Arntz, 2008; Roelofs, Huibers, Peeters, Arntz, & van Os, 2008). The current study extends these findings by showing that the same relation can be demonstrated by using a different measure focusing more specifically on the reactivation of ruminative thinking and that the relation remains robust even when neuroticism is measured several years before the assessment of rumination and depressive symptoms.

In those who had been depressed in the past, the relation between neuroticism and current symptoms of depression was also mediated by the ease with which hopelessness and suicidal thinking could be reactivated. The higher in neuroticism a previously depressed individual was, the more likely they were to respond to mild negative moods with reactivation of thoughts relating to hopelessness, or even suicidality, which, in turn, was related to current depressive symptoms. We suggest two potential explanations for why this factor mediated the relation between neuroticism and depression only in those who had been depressed in the past. According to differential activation theory (Teasdale, 1988), associations between negative patterns of thinking such as hopelessness and suicidal thinking and negative mood are particularly likely to be formed during previous depressive episodes and such learning might not have taken place in individuals who have not been depressed in the past suggesting that such patterns of thinking were unlikely to enter the pool of content that even frequent fluctuations in mood could have activated in this group. Furthermore, when feelings of hopelessness or suicidal thoughts occur, those who have been depressed in the past are likely to interpret them as more significant and as related to past failures to keep depression at bay possibly leading into rumination or avoidance while those who have never been depressed may appraise such thoughts as less dramatic and as part of a more fleeting experience. The fact that past history of depression had moderating effects both on the relation between neuroticism and cognitive reactivity (path *a*) and on the relation between cognitive reactivity and current symptoms of depression (path *b*) suggests that both of the above mechanisms may play a role in why reactivation of hopelessness and suicidal thinking occur as part of the causal path from neuroticism to depressive symptoms only in those with a previous history of depression.

Important limitations of the current study are that it is based completely on self-report and that previous history of depression was assessed using a self-report questionnaire for which only very limited information about psychometric characteristics was available. Cognitive reactivity is more commonly assessed using experimental designs and further research may use such methods to follow up the current results. However, the measure of cognitive reactivity used here has been shown to predict cognitive reactivity as assessed following mood induction in experimental studies (Van der Does, 2002). In particular, previous research has found that self-reports on hopelessness reactivity significantly predicted mood-related changes in a behavioural measure of hopelessness (Williams et al., 2008). The two LEIDS scales that emerged as significant mediators showed good internal consistency. However, for several other sub-scales, internal consistency was lower so reduced reliability might have contributed to the fact that these scales did not contribute significantly. Reactivation of ruminative tendencies and hopelessness only partially mediated the relation between neuroticism and current depression suggesting that other factors not assessed in the current study are likely to play a role in the causal mechanisms linking neuroticism and the occurrence of depressive symptoms. Furthermore it is not possible to rule out that third variables that were not assessed caused the observed relations.

In comparison to previous studies, the current study has the advantage of having assessed neuroticism as a predispositional factor several years before the assessment of current symptoms, thus, providing control against the effects of general reporting biases at the time of assessment, at least as far as the assessment of neuroticism is concerned. Unfortunately, the same is not true for the relation between cognitive reactivity and current symptoms of depression which were assessed at the same time. It is, therefore, possible that current symptoms of depression might have influenced ratings of cognitive reactivity, or neuroticism could have influenced both ratings of depression and cognitive reactivity without cognitive reactivity necessarily functioning as a mediator of the relation between neuroticism and depressive symptoms. Furthermore, while having measured the temperamental factor of neuroticism several years before assessment of cognitive reactivity makes the inference of causality more tenable, we cannot be sure whether one actually precedes the other. An important question in this regard relates to the extent to which observed relations may be due to conceptual overlap between constructs. Repetitive thinking, which is a hallmark of rumination, has been described as a cognitive manifestation of neuroticism, which assumes that this form of thinking is an integral aspect rather than a consequence of the construct. A look at item content shows that neuroticism scales include a number of items that include the term “worry”, a concept that is very close to rumination (de Jong-Meyer, Beck, & Riede, 2009) suggesting that the observed mediating effects of rumination may merely be a reflection of the fact that both neuroticism and rumination measures assess tendencies towards repetitive thinking and that such tendencies may remain relatively stable over time. However, when we re-analyzed our data using a neuroticism score that was based only on those items of the scale that did not explicitly relate to worry or cognitive reactivity and used numbers of symptoms at the time the neuroticism questionnaire was filled in as a covariate, results remained virtually unchanged. Results also remained virtually unchanged when these analyses were run with neuroticism scores corrected by additionally leaving out any items that referred to symptoms of depression. These findings seem to suggest that neuroticism encompasses a number of facets in addition to those directly referring to worry and repetitive thinking that seem to predispose individuals towards ruminative coping and increased cognitive reactivity. Results of mediator analyses also remained virtually unchanged when we controlled for overlap between BDI and LEIDS by taking out any items referring to rumination and suicidality from the BDI (items 3 and 9) and conducted analyses with the reduced BDI scale as dependent variable.

The current findings are in line with the notion that neuroticism represents a long term temperamental marker of risk for depression and suggest that this risk manifests and is mediated through increased tendencies to respond to negative mood with ruminative thinking and, in the case of those who have been depressed in the past, hopelessness and even suicidal thinking. Identifying factors that mediate the relation between neuroticism and depressive symptoms potentially provides more specific handles to therapeutically address the risk associated with this rather amorphous temperamental factor.

## Figures and Tables

**Table 1:** Mean scores (and standard deviations) on the EPQ neuroticism, BDI and LEIDS scales in participants with (*N* = 99) and without a previous history of depression (*N* = 83).

Measure	Previously depressed		Never depressed		*t*	*p*
	*M*	*SD*	*M*	*SD*		
EPQ Neuroticism	17.45	6.24	12.24	7.10	5.26	.00
BDI Depression	17.83	10.95	8.66	7.98	6.34	.00
LEIDS HOP	9.91	5.29	4.67	4.17	7.30	.00
LEIDS ACC	2.70	2.74	2.61	2.59	.22	*ns*
LEIDS AGG	9.26	5.18	6.61	4.24	3.72	.00
LEIDS CON	8.56	3.86	7.05	4.40	2.45	.02
LEIDS HAV	13.28	3.81	9.77	4.81	5.46	.00
LEIDS RUM	15.96	3.95	10.17	5.01	8.68	.00

LEIDS HOP = LEIDS Hopelessness/Suicidality, LEIDS ACC = LEIDS Acceptance/Coping, LEIDS AGG = LEIDS Aggression, LEIDS CON = LEIDS Control/Perfectionism, LEIDS HAV = LEIDS Harm Avoidance, LEIDS RUM = LEIDS Rumination.

**Table 2:** First-order correlations (*r*) between EPQ neuroticsm, BDI depression and LEIDS cognitive reactivity scores in participants with (*N* = 99) and without a previous history of depression (*N* = 83).

		Previously depressed								Never depressed							
		1	2	3	4	5	6	7	8	1	2	3	4	5	6	7	8
1.	EPQ Neuroticism	–								–							
2.	BDI Depression	.44**	–							.51**	–						
3.	LEIDS HOP	.40**	.57**	–						.49**	.40**	–					
4.	LEIDS ACC	.22*	.19	.25*	–					.19	.21	.19	–				
5.	LEIDS AGG	.30**	.19	.41**	.15	–				.40**	.33**	.41**	.19	–			
6.	LEIDS CON	.24*	.18	.25*	.19	.27**	–			.38*	.27*	.44**	.59**	.27*	–		
7.	LEIDS HAV	.31**	.34**	.38**	.26*	.05	.39**	–		.46**	.42**	.59**	.41**	.29**	.41**	–	
8.	LEIDS RUM	.31**	.42**	.49**	.10	.35**	.19	.51**	–	.51**	.55**	.62**	.23*	.45**	.42**	.73**	–

LEIDS HOP = LEIDS Hopelessness/Suicidality, LEIDS ACC = LEIDS Acceptance/Coping, LEIDS AGG = LEIDS Aggression, LEIDS CON = LEIDS Control/Perfectionism, LEIDS HAV = LEIDS Harm Avoidance, LEIDS RUM = LEIDS Rumination.

**Table 3:** Summary of multiple mediator model analyses in participants with (*N* = 99) and without (*N* = 83) a previous history of depression (5000 bootstraps).

	Independent variable	Mediating variable	Dependent variable	Effect of IV on M	Effect of M on DV	Direct Effect	Indirect Effect		Total Effect
	(IV)	(M)	(DV)	(a)	(b)	(*c*′)	(a × b)	95% CI	(c)
Previously Depressed	EPQ-N	LEIDS HOP	BDI	.33**	.78**	.38*	.26*	(.11–.50)	.72**
		LEIDS ACC		.09*	.22		.02	(−.03–.11)	
		LEIDS AGG		.24**	−.26		−.06	(−.21–.03)	
		LEIDS CON		.15*	.07		.01	(−.07–.12)	
		LEIDS HAV		.18**	−.10		−.02	(−.17–.07)	
		LEIDS RUM		.19**	.64*		.13*	(.02–.32)	

Never depressed	EPQ-N	LEIDS HOP	BDI	.28**	.03	.35*	.01	(−.14–.14)	.57**
		LEIDS ACC		.06	.38		.02	(−.01–.16)	
		LEIDS AGG		.23**	.05		.01	(−.10–.15)	
		LEIDS CON		.24**	−.16		−.04	(−.16–.05)	
		LEIDS HAV		.31**	−.11		−.03	(−.20–.11)	
		LEIDS RUM		.36**	.68**		.24*	(.07–.52)	

EPQ-N = EPQ Neuroticism, BDI = BDI Depression Sumscore, LEIDS HOP = LEIDS Hopelessness/Suicidality, LEIDS ACC = LEIDS Acceptance/Coping, LEIDS AGG = LEIDS Aggression, LEIDS CON = LEIDS Control/Perfectionism, LEIDS HAV = LEIDS Harm Avoidance, LEIDS RUM = LEIDS Rumination.
